# Capillary Refill Time as a Part of Routine Physical Examination in Critically Ill Patients Undergoing Vasoactive Therapy: A Prospective Study

**DOI:** 10.3390/jcm13195782

**Published:** 2024-09-28

**Authors:** Fabian Wesołek, Zbigniew Putowski, Wiktoria Staniszewska, Robert Latacz, Łukasz J. Krzych

**Affiliations:** 1 Students’ Scientific Society, Department of Acute Medicine, School of Medicine in Zabrze, Medical University of Silesia, 40-055 Katowice, Poland; wiktoria.staniszewska@gmail.com (W.S.); robert_latacz@o2.pl (R.L.); 2 Center for Intensive and Perioperative Care, Jagiellonian University Medical College, 31-008 Krakow, Poland; putowski.zbigniew@gmail.com; 3 Department of Acute Medicine, School of Medicine in Zabrze, Medical University of Silesia, 40-055 Katowice, Poland; lkrzych@sum.edu.pl; 4 Department of Cardiac Anaesthesiology and Intensive Care, Silesian Centre for Heart Diseases, 41-800 Zabrze, Poland

**Keywords:** blood pressure, peripheral perfusion, vasoactive agents, hemodynamics

## Abstract

**Background/Objectives:** In critically ill patients, achieving a mean arterial pressure (MAP) of 65 mmHg is a recommended resuscitation goal to ensure proper tissue oxygenation. Unfortunately, some patients do not benefit from providing such a value, suggesting that other indices are needed for better hemodynamic assessment. Capillary refill time (CRT) has emerged as an established marker for peripheral perfusion and a therapeutic target in critical illness, but its relationship with other exponents of hypoperfusion during vasopressor support after resuscitation period still warrants further research. This study aimed to investigate whether in critically ill patients after initial resuscitation, CRT would provide information independent of other, readily accessible hemodynamic variables. **Methods:** Critically ill patients who were mechanically ventilated after the resuscitation period and receiving vasopressors were prospectively studied between December 2022 and June 2023. Vasopressor support was measured using norepinephrine equivalent doses (NEDs). CRT, MAP and NED were assessed simultaneously and analyzed using Spearman’s rank correlation. **Results:** A total of 92 patients were included and 210 combined MAP-CRT-NED-Lactate records were obtained. There was no correlation between CRT and MAP (R = −0.1, *p* = 0.14) or lactate (R = 0.11, *p* = 0.13), but there was a positive weak correlation between CRT and NED (R = 0.25, *p* = 0.0005). In patients with hypotension, in 83% of cases (15/18), CRT was within normal range, despite different doses of catecholamines. When assessing patients with high catecholamine doses, in 58% cases (11/19), CRT was normal and MAP was usually above 65 mmHg. **Conclusions:** Capillary refill time provides additional hemodynamic information that is not highly related with the values of mean arterial pressure, lactate level and vasopressor doses. It could be incorporated into routine physical examination in critically ill patients who are beyond initial resuscitation.

## 1. Introduction

Hemodynamically unstable patients require prompt and adequate treatment to ensure reperfusion of hypoxic tissues. To ensure this, complex hemodynamic monitoring is employed and goal-directed therapy is performed. For example, in the Surviving Sepsis Campaign (SSC) guidelines, there is a strong recommendation that the initial target for mean arterial pressure (MAP) in septic shock should be at least 65 mmHg [1,2]. It is based on the assumption that such pressure should sustain blood flow to vital organs by increasing perfusion pressure above the lower limit of auto-regulation curves [3,4]. However, 79% of septic shock patients have MAP >65 mmHg within the first 3 h of septic shock resuscitation [5], and yet 40% of these patients continue to develop multi-organ failure and ultimately die [2]. This may indicate that MAP is not an ideal resuscitation target and other perfusion indicators are employed, for example, lactate concentration or capillary refill time (CRT). CRT is an indicator of skin perfusion that can be easily and frequently performed, and can be particularly utilized in resource-limited settings [6]. It has been shown to correlate with dynamic organ perfusion changes in response to resuscitation [7,8]. Moreover, CRT has been proven to hold a prognostic value for mortality [7,9,10] and its use as a resuscitation target has been shown to be superior to lactate concentration [11]. Hence, CRT provides valuable and independent information about the hemodynamic state of a patient during the initial resuscitation phase.

In critically ill patients undergoing vasoactive treatment who are in the post-resuscitation period, the role of skin perfusion assessment is limited, as it is no longer a direct indicator of dynamic reperfusion. In this study, we hypothesized that in critically ill patients after initial resuscitation, CRT would provide information independent of other, readily accessible hemodynamic variables.

## 2. Material and Methods

This prospective, single-center, observational study was performed in the intensive care unit (ICU) of a tertiary university hospital in Poland between December 2022 and June 2023. 

Of all the consecutive patients admitted to the ICU, those who (1) were mechanically ventilated, (2) underwent vasoactive treatment, (3) had an invasive blood pressure measurement, (4) were over the age of 18 and (5) were in the post-resuscitation period were eligible for the study inclusion. Post-resuscitation period was regarded as a period after initial resuscitation, in which a patient was not dynamically treated with fluids, due to either fluid unresponsiveness (pulse pressure variation <13%) or physician’s judgement not to further administer fluids. Moreover, at the point of observation, vasoactive drug doses were not actively titrated. Patients with an inaccessible distal phalanx of the finger and patients in a room with suboptimal room temperature were excluded from the study. An inaccessible distal phalanx was defined as any condition that impaired accurate CRT assessment, such as severe peripheral edema, amputation or severe skin condition. Suboptimal room temperature was defined as a temperature significantly different from the standard ICU environment, potentially affecting peripheral perfusion.

The objective of the study was to describe a relationship between the assessment of peripheral perfusion (via CRT) and other, readily available hemodynamic variables, namely MAP, lactate concentration and norepinephrine-equivalent doses (NEDs). It was assumed that incorporating CRT measurements during routine physical examinations would provide independent information (hence, the variables would not be highly correlated).

The study involved simultaneous measurement of CRT, MAP, lactate concentration and the dose of vasopressors. CRT was measured using a standardized technique to ensure good inter-rater reliability [5,12]. The CRT measurement consisted of pressing the index finger of the right hand with a laboratory slide until the skin blanches, then holding the pressure for 10 s. The duration it took for the skin to regain its color was measured using a chronometer, and CRT > 3 s was considered abnormal. To standardize the light, an iPhone flashlight was used. Standardization of the CRT measurement included training sessions for all staff involved in the study to minimize variability between observers. Each observer practiced the technique on a series of study subjects to ensure consistency. The measurement of MAP was conducted invasively using an arterial catheter inserted into a suitable artery, usually the radial artery. Lactate concentration was measured within 3 h of CRT assessment. Lactate > 2 mmol/L was considered pathological. The type, flow and dilution of vasopressor drugs were recorded and measured as norepinephrine equivalent doses (NEDs). An NED quantifies the total amount of vasopressors, considering the potency of each such agent, which typically includes catecholamines, derivatives and vasopressin [13]. NED > 0.5 μg/kg/min was considered a “high” dose of vasoactive drugs. The set of measurements (CRT, MAP, lactates and NEDs) were taken up to five times from a single patient but with at least a 24 h gap between each set.

The study was approved by the Bioethics Committee of the Medical University of Silesia (PCN/CBN/0052/KB/242/22, approval date: 7 December 2022). All participants agreed to participate in the study. The STROBE (Strengthening the Reporting of Observational Studies in Epidemiology) statement was applied for appropriate reporting [14].

Due to the exploratory nature of the study, no sample size calculation was performed. It was assumed that the results should be treated as hypothesis-generating. All analyses were performed using R version 4.2.0 (RProject). Continuous variables were expressed as median and interquartile range (IQR). Qualitative variables were expressed as absolute values and/or percentages. The relationship between CRT and MAP/NEE/lactate was evaluated using the Spearman’s rank correlation coefficient. A *p*-value < 0.05 was considered statistically significant.

## 3. Results

**Figure 1:**
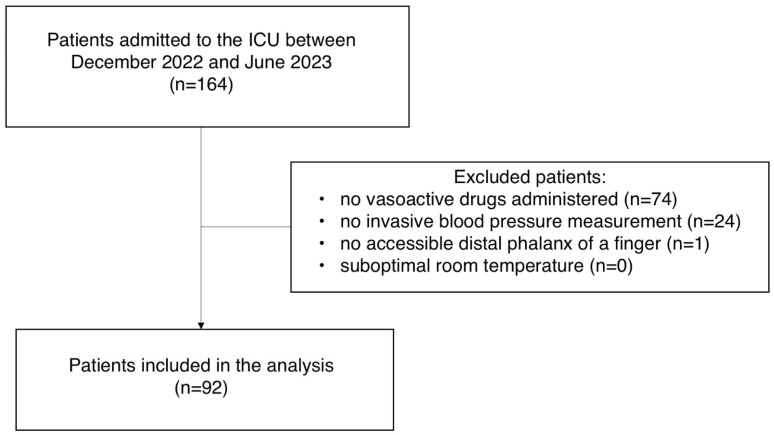
Flow chart of study population. ICU—intensive care unit.

A total of 92 patients were included (Figure 1). We obtained 210 combined MAP-CRT-NED-Lactate records. The median age of the patients was 66 years (45–72). In 165 records (78.6%), CRT was within the normal range, with the median of 1.9 sec (IQR 1.25–2.75). The median MAP was 82 mmHg (74–90). The median lactate level was 2.1 mmol/L (1.46–3.1). The median NED was 0.1 μg/kg/min (0.04–0.20). The ICU mortality was 39 (42%). Acute respiratory failure (34.8%), subarachnoid hemorrhage (SAH) (22.8%) and acute neurological diseases other than SAH (18.5%) predominated as the reason for admission. CRT values were not prognostically associated with mortality (1.63 vs. 1.955 sec; *p* = 0.13). Detailed demographic and clinical characteristics are shown in Table 1.

**Figure 2:**
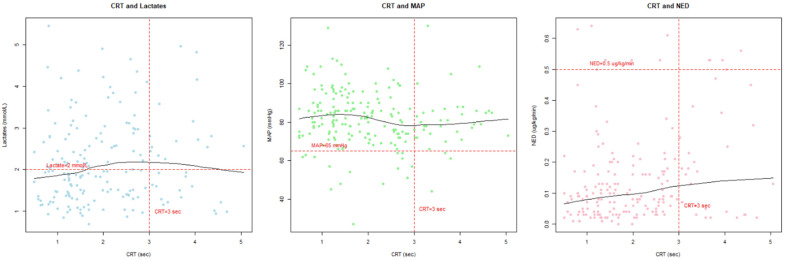
Scatter plots with smoothed lines depicting relationship between capillary refill time and mean arterial pressure, norepinephrine equivalent dose and lactate level (10% outliers removed). CRT—capillary refill time; MAP—mean arterial pressure; NED—norepinephrine equivalent dose.

There was a very wide dispersion of CRT values for their corresponding MAP, NED and lactate concentrations (Figure 2). There was no correlation between CRT and MAP (r = −0.1, *p* = 0.14), and CRT and lactate (r = 0.11, *p* = 0.13), but there was a positive weak correlation between CRT and NED (r = 0.25, *p* = 0.0005).

**Figure 3:**
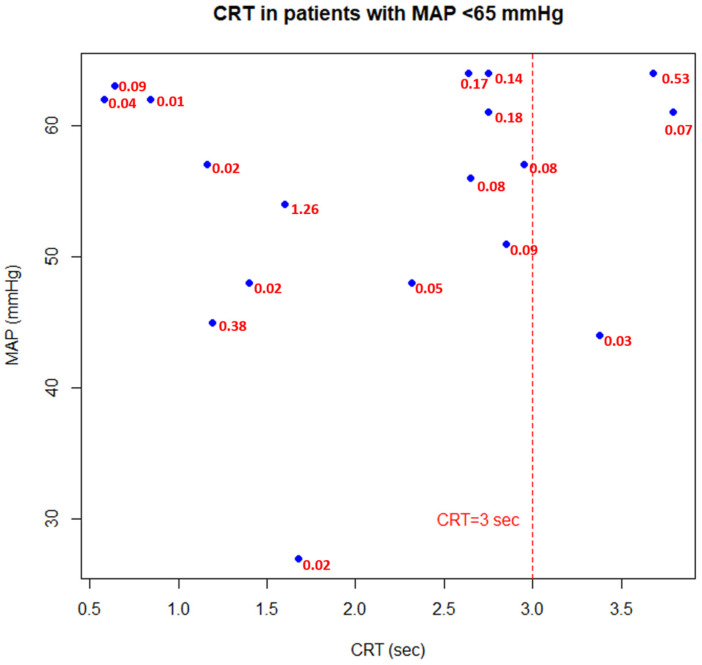
Capillary refill time in patients with mean arterial pressure <65 mmHg (hypotension). Red values correspond to norepinephrine equivalent doses (ug/kg/min). CRT—capillary refill time; MAP—mean arterial pressure.

**Figure 4:**
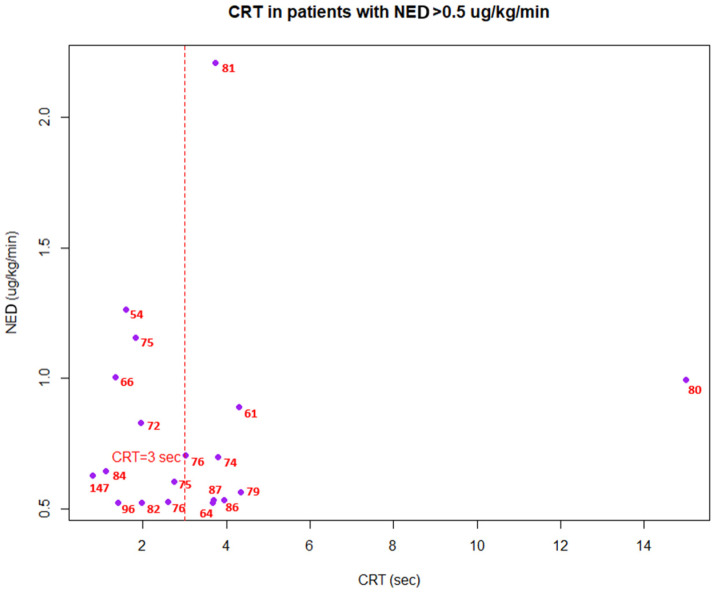
Capillary refill time in patients with norepinephrine equivalent doses >0.5 ug/kg/min (high catecholamine doses). Red values correspond to mean arterial pressure (mmHg). CRT—capillary refill time; NED—norepinephrine equivalent dose.

In patients with hypotension (i.e., MAP < 65 mmHg), in 83% of cases (15/18), CRT was within a normal range (i.e., 0–3 sec), despite a range of different doses of catecholamines (Figure 3). When assessing patients with high catecholamine doses (i.e., NED > 0.5 ug/kg/min), in 58% cases (11/19), CRT was considered normal and MAP was usually above 65 mmHg (Figure 4).

## 4. Discussion

The main goal of this study was to determine whether in critically ill patients after initial resuscitation, CRT would provide information independent of other, readily accessible hemodynamic variables. We found that CRT was fairly independent of MAP, lactate concentration and catecholamine doses. Additionally, CRT was rarely prolonged during hypotension (MAP < 65 mmHg) and was often considered normal (<3 sec) during administration of high doses of catecholamines (NED > 0.5 μg/kg/min).

CRT has been reported as a reliable indicator of dynamic perfusion changes during the resuscitation phase in different types of shock. For example, Jacquet-Lagrèze et al. showed that CRT in combination with passive leg raising (PLR) is able to predict peripheral perfusion response to fluids in patients in circulatory shock [15]. Furthermore, Raia et al. observed significant CRT dynamics after administering 500 mL of crystalloids in patients with sepsis, in which fluctuations greater than 0.2 s were considered significant and were observed 6–8 min after the start of fluid therapy [16]. These studies highlighted CRT’s high sensitivity to preload changes. However, in patients undergoing vasoactive treatment, often days after initial resuscitation, the interpretation of CRT values appears to be more complicated. A study by Hernandez et al. tested the CRT response to norepinephrine dose uptitration (transient MAP increase from 67 to 84 mmHg) in patients with septic shock after initial resuscitation [17]. It was found that increasing norepinephrine produced various CRT changes, including positive, neutral and negative responses. While 14 out of 25 patients exhibited CRT decrease after a raise in MAP, 4 patients had their CRT prolonged, which further nuanced distinct behavior of peripheral circulation.

Our study, compared to the above, did not focus on the variability of CRT against hemodynamic intervention, but on the independence of CRT against hemodynamic parameters. Firstly, higher MAP levels were not associated with better CRT values, suggesting that normal macrohemodynamics are not linearly associated with improved peripheral perfusion and microcirculation, suggesting a rather personalized response to MAP levels [18]. Moreover, we found that in cases with MAP <65 mmHg (Figure 3), CRT was almost always within normal range, which points that peripheral perfusion can be maintained despite lower MAP targets (≤ 65 mmHg), highlighting the potential of CRT as an independent indicator in situations where conventional hemodynamic targets may not be achieved. Furthermore, lactate levels were dissociated from the values of CRT. It could be speculated that stimulation of anaerobic metabolism may occur independently of changes in peripheral perfusion and reverse. This finding appears to be relevant, as in patients in newly diagnosed, acute circulatory dysfunction, CRT prolongation and hyperlactatemia often coexist [19]. We additionally found that there is a low but positive correlation between CRT and NEDs. Such a finding could be interpreted in two ways. Firstly, increasing NEDs may be associated with excessive vasoconstriction [20] and tissue hypoperfusion (thus, prolonged CRT), or, secondly, patients with a greater degree of microcirculatory dysfunction require greater NEDs (hence, a positive correlation between CRT and NEDs). However, as CRT was not well correlated with lactates, the first hypothesis seems to be more reasonable. It remains to be determined whether reliable CRT assessment is confounded by vasoconstrictive effects of vasoactive agents. Despite this finding, it was found that in cases with high vasopressor doses (NEDs > 0.5 ug/kg/min), the majority of patients (58%) had a normal CRT, which indicates that even with high doses of catecholamines, they may not induce an excessive vasoconstriction of the skin vascular bed. Lastly, CRT values were not prognostically associated with mortality in this mixed-ICU population, highlighting a non-obvious interpretation of CRT values in patients after resuscitation period.

Strengths of this study include the following: the inclusion of patients with invasive blood pressure measurements, the use of standardized CRT measurement technique and the dose synthesis of vasoactive drugs by using the NED. The study has several weaknesses; even though a standardized method was used to obtain these measurements, they are still subjected to human error and there are factors that interfere with its measurement, such as gender, age, lighting, ambient temperature, place of measurement and the amount of pressure applied [21,22,23,24]. Secondly, this was a single-center study of purely observational design which limited the interpretation of CRT values. Thirdly, to increase the number of measurements, we allowed for multiple (up to five with at least a one-day interval) assessments of single patients, which may have led to sampling bias. In addition, due to a lack of human resources, data were not collected every day, resulting in a small study sample. Moreover, the study group was heterogeneous in terms of the type and reason of hemodynamic support, but deterioration of chronic heart failure was the cause of treatment in only two patients. Future studies could focus on subgroup analysis to determine whether specific patient populations show different CRT responses.

## 5. Conclusions

Capillary refill time provides additional hemodynamic information that is not highly related with the values of mean arterial pressure, lactate level and vasopressor doses. It could be incorporated into routine physical examination in critically ill patients who are beyond initial resuscitation. The relevance of these results remains to be investigated.

## Figures and Tables

**Table 1:** Demographic and clinical characteristics.

Characteristics	All Patients (n = 92)
Age—median (IQR), years	66 (45–72)
Gender—n (%)	
Female	45 (49%)
Male	47 (51%)
CRT—median (IQR), s	1.9 (1.25–2.75)
MAP—median (IQR), mmHg	82 (74–90)
Lactate—median (IQR), mmol/L	2.1 (1.46–3.1)
NED—median (IQR), μg/kg/min	0.01 (0.04–0.20)
ICU mortality—n (%)	39 (42%)
Cause of admission—n(%)	
Acute respiratory failure	32 (34.8%)
Subarachnoid hemorrhage (SAH)	21 (22.8%)
Acute neurological disease (other than SAH)	17 (18.5%)
Acute pancreatitis	5 (5.4%)
Sudden cardiac arrest	4 (4.3%)
Acute heart failure	3 (3.3%)
Septic shock	3 (3.3%)
Shock (other than septic)	3 (3.3%)
Acute liver failure	2 (2.2%)
Malignant neoplasm	2 (2.2%)

## Data Availability

The datasets used and analyzed during the current study are available from the corresponding author on reasonable request.

## Abbreviations

MAP	Mean Arterial Pressure
CRT	Capillary Refill Time
NED	Norepinephrine Equivalent Dose
SSC	Surviving Sepsis Campaign
ICU	Intensive Care Unit
STROBE	Strengthening the Reporting of Observational Studies in Epidemiology
IQR	Interquartile Range
SAH	Subarachnoid Hemorrhage
PLR	Passive Leg Raising
